# *Preventing Chronic Disease*: Moving Forward in 2011

**Published:** 2010-12-15

**Authors:** Samuel F. Posner

*Preventing Chronic Disease* (*PCD*) had a very exciting and busy year. In January 2010, we outlined changes and enhancements to implement during the year. It is rewarding to report that these advances are being developed or have been implemented successfully. Following the path of leadership set by *PCD’s* founding editor, Dr Lynne Wilcox, *PCD* is dedicated to innovations that improve the quality of the journal. None of these changes would have been possible without the enthusiastic commitment of the journal’s staff.

We have instituted several visible changes, the most prominent of which was increasing publication from 4 to 6 issues per year. Among the benefits of the schedule change was a decline in the time from acceptance to publication — from 1 year to approximately 7 months. We hope to continue to shorten this time in the coming year by increasing production efficiency without compromising quality. Another visible change in 2010 has been the introduction of *PCD* Dialogue, which gives readers the opportunity to engage in discussions of selected *PCD* articles.

The number of submissions to *PCD* has also increased by 20% from last year. To broaden our reach, we continue to encourage public health professionals to submit to *PCD* through professional listserves, conferences, and other networks. Current examples include a call for student author submissions and a call for submissions on the health of veterans. This combination of increased submissions and efforts to decrease time to publication has resulted in a decline in the acceptance rate during the past few years.

Historically, each issue of *PCD* centered on a specific theme coordinated by a guest editor. In 2010, we focused on publishing regular submissions, although we continued to publish collections of articles on particular themes from time to time. For example, the July, September, and November 2010 issues included collections of papers from the Mobilizing Action Toward Community Health (MATCH) project. In the future, we expect to see more special collections published as part of a regular issue. Another landmark in *PCD* publishing is this issue, which includes 6 articles that are copublished by *PCD* and *Chronic Diseases in Canada*. This effort reflects collaboration among authors and staff from both journals to publish a common set of papers, which will increase the reach of both journals.

We are tracking several indicators to better understand and serve our readership. The increased number of visits to and views of the *PCD* website is evidence that we are increasing our reach and publishing articles of interest to those working in the field. The direct feedback from the field about the relevance and quality of *PCD* is another important gauge of our impact. I have received direct feedback from colleagues in health departments, community-based organizations, and academic institutions on how valuable *PCD* is to their work. One of the nation’s larger health care systems provided reprints of the MATCH papers from our July and September issues to all of its senior hospital executives at their annual meeting. Hospital executives do not immediately come to mind when considering *PCD's* reach; however, their interest demonstrates a recognition of the importance of integrating public health and health care and the relevance of *PCD* content to a variety of audiences.

The editorial board is a critical part of the success of *PCD*. This year, we instituted some changes in the board structure. First, we implemented term limits on board membership. Having a longstanding, consistent board has been necessary to establish and grow the journal; however, we are now at a point at which we can give these founding board members time to rest and allow new people to contribute. *PCD* is forever in debt to Drs Laura Kann, Matt Kreuter, John Kurata, Rose Martinez, Lucero Rodriguez, and Linda Wright for their contributions to the board and their commitment and dedication to *PCD*. We owe the same debt to Drs Barbara Bowman, Ross Brownson, David Fleming, Jeff Harris, Shiriki Kumanyika, Christopher Maylahn, Mark Messonnier, Nico Pronk, Patrick Remington, Barbara Riley, and Martina Taylor for staying with us for another few years. *PCD* welcomes our incoming board members Drs Ralph Fuccillo, Sara Huston, Sherman James, Eugene Lengerich, Susan Meikle, and Joel Moskowitz. All of these new board members are leaders in their fields as both researchers and practitioners, and *PCD* is grateful for the opportunity to benefit from their expertise.

In 2011, we will be working on several additional innovations, including improving our production processes, providing articles in an e-reader format, and preparing to change to a rolling publication schedule. We will be expanding *PCD* Dialogue to include more articles in each issue and hope to increase the use of this tool by our readership. Many of the changes in our production process will not be obvious to the readers of *PCD*; however, the authors should see a further reduction in time to publication and a reduced editing and proofing workload. As technology develops, *PCD* is working to adapt and integrate new technologies into our publishing enterprise. Many people now read their books, newspapers, and even scholarly journals by using e-readers. Although we already provide courtesy copies of journal articles as PDFs, which can be printed, a growing number of readers, particularly students, work in a virtually paperless environment. E-reader technology prepares *PCD* for that changing research dynamic and keeps us ahead of the curve in innovation.

As editor in chief, I have enjoyed the challenges and rewards of leading *PCD*. My job is especially rewarding because I work with an outstanding team of editors, production staff, administrative professionals, and an editorial board who make the internal workings run smoothly. At the same time, we all owe a debt of gratitude to the reviewers who provide thoughtful reviews of our submissions and make our editorial decisions much easier. Finally, without the readers and authors of *PCD*, the journal would not exist. The entire staff of *PCD* thanks you for your contributions and for participating in the success of the journal. This has been a highly productive year for *PCD*. We look forward to a productive 2011, and we will continue working to maintain and increase the high quality standards of *PCD*.

